# Effect of treatment periods on efficacy of glecaprevir and pibrentasvir in chronic hepatitis C: A nationwide, prospective, multicenter study

**DOI:** 10.1002/jgh3.13068

**Published:** 2024-04-25

**Authors:** Atsuhiro Morita, Nobuharu Tamaki, Haruhiko Kobashi, Nami Mori, Keiji Tsuji, Shintaro Takaki, Chitomi Hasebe, Takehiro Akahane, Hironori Ochi, Toshie Mashiba, Naohito Urawa, Hideki Fujii, Akeri Mitsuda, Masahiko Kondo, Chikara Ogawa, Yasushi Uchida, Ryoichi Narita, Hiroyuki Marusawa, Yoshihito Kubotsu, Tomomichi Matsushita, Masaya Shigeno, Hideo Yoshida, Katsuaki Tanaka, Eisuke Okamoto, Toyotaka Kasai, Toru Ishii, Kazuhiko Okada, Masayuki Kurosaki, Namiki Izumi

**Affiliations:** ^1^ Department of Gastroenterology Japanese Red Cross Kyoto Daini Hospital Kyoto Japan; ^2^ Department of Gastroenterology and Hepatology Musashino Red Cross Hospital Tokyo Japan; ^3^ Department of Gastroenterology Japanese Red Cross Okayama Hospital Okayama Japan; ^4^ Department of Gastroenterology Hiroshima Red Cross Hospital & Atomic‐bomb Survivors Hospital Hiroshima Japan; ^5^ Department of Gastroenterology Japanese Red Cross Asahikawa Hospital Asahikawa Japan; ^6^ Department of Gastroenterology Ishinomaki Red Cross Hospital Ishinomaki Japan; ^7^ Center for Liver‐Biliary‐Pancreatic Disease Matsuyama Red Cross Hospital Matsuyama Japan; ^8^ Department of Gastroenterology and Hepatology Ise Red Cross Hospital Ise Japan; ^9^ Department of Gastroenterology Japanese Red Cross Kyoto Daiichi Hospital Kyoto Japan; ^10^ Department of Gastroenterology Tottori Red Cross Hospital Tottori Japan; ^11^ Department of Gastroenterology Otsu Red Cross Hospital Otsu Japan; ^12^ Department of Gastroenterology and Hepatology Takamatsu Red Cross Hospital Takamatsu Japan; ^13^ Department of Gastroenterology Matsue Red Cross Hospital Matsue Japan; ^14^ Department of Gastroenterology Oita Red Cross Hospital Oita Japan; ^15^ Department of Gastroenterology and Hepatology Osaka Red Cross Hospital Osaka Japan; ^16^ Department of Internal Medicine Karatsu Red Cross Hospital Saga Japan; ^17^ Department of Gastroenterology Japanese Red Cross Gifu Hospital Gifu Japan; ^18^ Department of Gastroenterology Japanese Red Cross Nagasaki Genbaku Hospital Nagasaki Japan; ^19^ Department of Gastroenterology Japanese Red Cross Medical Center Tokyo Japan; ^20^ Department of Gastroenterology Hatano Red Cross Hospital Hatano Japan; ^21^ Department of Gastroenterology Masuda Red Cross Hospital Masuda Japan; ^22^ Department of Gastroenterology Fukaya Red Cross Hospital Saitama Japan; ^23^ Department of Gastroenterology Japanese Red Cross Akita Hospital Akita Japan; ^24^ Department of Gastroenterology Toyama Red Cross Hospital Toyama Japan

**Keywords:** chronic hepatitis C, cirrhosis, glecaprevir and pibrentasvir (GLE/PIB), treatment periods

## Abstract

**Background and aim:**

In patients with chronic hepatitis C, 8 weeks of glecaprevir and pibrentasvir (GLE/PIB) treatment for chronic hepatitis (non‐cirrhosis) and 12 weeks for cirrhosis have been approved in Japan. However, whether 8 weeks of treatment for cirrhosis may reduce treatment efficacy has not been adequately investigated.

**Methods:**

This prospective, nationwide, multicenter cohort study enrolled 1275 patients with chronic hepatitis C who received GLE/PIB therapy. The effect of liver fibrosis and treatment periods on the efficiency of GLE/PIB therapy was investigated. The primary endpoint was the sustained virological response (SVR) rate in patients with chronic hepatitis (non‐cirrhosis) and cirrhosis. The association between treatment periods and liver fibrosis on the SVR after 12 weeks of treatment rate was investigated.

**Results:**

The SVR rates in patients with chronic hepatitis with 8 weeks of treatment, chronic hepatitis with 12 weeks of treatment, cirrhosis with 8 weeks of treatment, and cirrhosis with 12 weeks of treatment were 98.9% (800/809), 100% (87/87), 100% (166/166), and 99.1% (211/213), respectively, and were was not different among these groups (*P* = 0.4).

**Conclusion:**

GLE/PIB therapy for chronic hepatitis C had high efficacy regardless of liver fibrosis status and treatment periods. Periods of GLE/PIB therapy could be chosen with available modalities, and high SVR rates could be achieved regardless of the decision.

## Graphical abstract

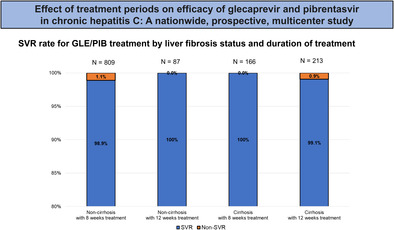

## Introduction

More than 100 million people in Japan have hepatitis C virus (HCV) infection.^1^ Since chronic hepatitis C can lead to liver cirrhosis and hepatocellular carcinoma (HCC), HCV elimination is important to prevent the progression to liver disease.^2^, ^3^, ^4^, ^5^, ^6^, ^7^ The development of newer direct‐acting antiviral agents (DAAs) for HCV has improved the rate of sustained virological response (SVR), and DAA therapy has expanded the indication for antiviral therapy.^8^, ^9^, ^10^, ^11^, ^12^, ^13^

Recently, new interferon‐free DAA regimens have become available, including glecaprevir (GLE) and pibrentasvir (PIB). GLE/PIB therapy has high anti‐HCV activity and is effective against pan‐genotype HCV.^14^, ^15^, ^16^, ^17^, ^18^ Japanese clinical trials of this GLE/PIB regimen (CERTAIN‐1 and 2 trials) have shown a high response rate in Japanese patients who had DAA‐naive HCV infection with genotype 1 and 2 infections.^19^, ^20^ Based on the result, GLE/PIB therapy has been approved in Japan and recommended as the first‐line treatment in the Japan Society of Hepatology (JSH) guideline.^21^ In Japan, 8 weeks of treatment with GLE/PIB for chronic hepatitis (non‐cirrhosis) and 12 weeks of treatment for cirrhosis have been approved. However, in clinical practice, liver fibrosis may be difficult to assess accurately, and an appropriate treatment period may not be selected. Specifically, whether 8 weeks of treatment for cirrhosis may reduce treatment efficacy has not been adequately investigated. To fill the current gap in knowledge, we investigated the effect of liver fibrosis and treatment periods on the efficiency of GLE/PIB therapy using a nationwide, multicenter, prospective cohort.

## Methods

### Study protocol

This prospective, nationwide, multicenter cohort study, assembled by the Japanese Red Cross Liver Study Group, enrolled 1687 patients with chronic hepatitis C who received GLE/PIB therapy. Patients were recruited from 24 participating sites across the country between November 2017 and January 2019. The exclusion criteria were as follows: (i) previous history of DAA therapy, (ii) patients who were not evaluated for SVR after 12 weeks of treatment (SVR12), and (iii) loss to follow‐up or incomplete treatment. Since GLE/PIB therapy is not approved for decompensated cirrhosis in Japan, we ensured no patient had decompensated cirrhosis. After excluding these patients, 1275 patients with chronic hepatitis C were included in the study (Fig. S1). The efficacy of 8 weeks of treatment for chronic hepatitis (non‐cirrhosis) and 12 weeks of treatment for cirrhosis was evaluated.

Treatment periods (8 or 12 weeks) were chosen based on the decision of each physician. Informed consent was obtained from each patient. This study was approved by the ethics committee of each participating hospital associated with the Japanese Red Cross and conformed to the ethical principles of the 1975 Declaration of Helsinki.

### Laboratory and virologic assessments

HCV‐RNA levels were measured using the COBAS Taq‐Man HCV test (Roche Diagnostics, Tokyo, Japan). SVR12 was evaluated in all patients and used as the primary endpoint. The HCV genotype was determined by sequence determination in the 5′ nonstructural region of the HCV genome, which was followed by phylogenic analysis.

### Liver fibrosis assessment

Apart from the clinical diagnoses made by each physician, liver fibrosis was assessed retrospectively for the analyses of the study. The Fibrosis‐4 (FIB‐4) index was used to assess liver fibrosis at baseline. FIB‐4 >3.25 was used as the threshold of cirrhosis.^22^, ^23^

### Primary endpoint

The primary endpoint was the SVR12 rate in patients with chronic hepatitis (non‐cirrhosis) and cirrhosis. The association between treatment periods and liver fibrosis on the SVR rate was investigated.

### Statistical analysis

Patient characteristics were compared using the Mann–Whitney U test for continuous variables or Fisher's exact test for categorical variables. The SVR12 rate among groups was compared using the Cochran–Armitage test. Statistical significance was defined as *P* < 0.05 in all analyses. SPSS version 2 was used in the statistical analysis.

## Results

### Patient characteristics and SVR rate

The study enrolled a total of 1275 patients who received GLE/PIB therapy. The baseline patient characteristics are shown in Table 1. The median age was 66 years, and 54.3% were females. Among the patients, 5.8% had a history of HCC. The proportions of patients with genotypes 1 and 2 were 39.9% and 41.9%, respectively. Patients who received 12 weeks of treatment were significantly older and had higher levels of aspartate aminotransferase (AST), alanine aminotransferase (ALT), and FIB‐4, and lower albumin levels and platelet count than patients who received 8 weeks of treatment. In all patients, the SVR rate of GLE/PIB therapy was 99.1% (1264/1275).

**Table 1:** Patient characteristics

	All patients	8 weeks of treatment	12 weeks of treatment	
Number	1275	975	300	*P*‐value
Age, years	66 (55–75)	64 (54–73)	72 (64–80)	<0.001
Gender, %				
Female	692 (54.3)	539 (55.3)	153 (51.0)	0.2
Male	583 (45.7)	436 (44.7)	147 (49.0)	
Genotype, %				
1	509 (39.9)	390 (40.0)	119 (39.7)	0.008
2	534 (41.9)	410 (42.1)	124 (41.3)	
Others	16 (1.3)	6 (0.6)	10 (3.3)	
ND	216 (16.9)	169 (17.3)	47 (15.7)	
History of HCC, %	74 (5.8)	11 (1.1)	63 (21.0)	<0.001
Albumin, g/dL	4.1 (3.8–4.3)	4.1 (3.9–4.4)	3.8 (3.5–4.1)	<0.001
AST, IU/L	34 (24–54)	32 (24–48)	47 (29–71)	<0.001
ALT, IU/L	32 (20–58)	30 (19–54)	39 (21–67)	0.001
Platelet counts, ×10^4^/μL	18.0 (14–23)	19.4 (16–24)	12 (9.1–15)	<0.001
HCV‐RNA, log IU/mL	6.3 (5.5–6.7)	6.3 (5.6–6.7)	6.1 (5.4–6.5)	0.001
FIB‐4	2.19 (1.43–3.69)	1.90 (1.28–2.73)	4.43 (3.04–6.39)	<0.001

*P*‐value indicates the comparison between 8 weeks and 12 weeks of treatment.

Continuous data are shown in median (interquartile range).

ALT, alanine aminotransferase; AST, aspartate aminotransferase; HCC, hepatocellular carcinoma; ND, not determined.

### Effect of genotype on SVR

**Figure 1:**
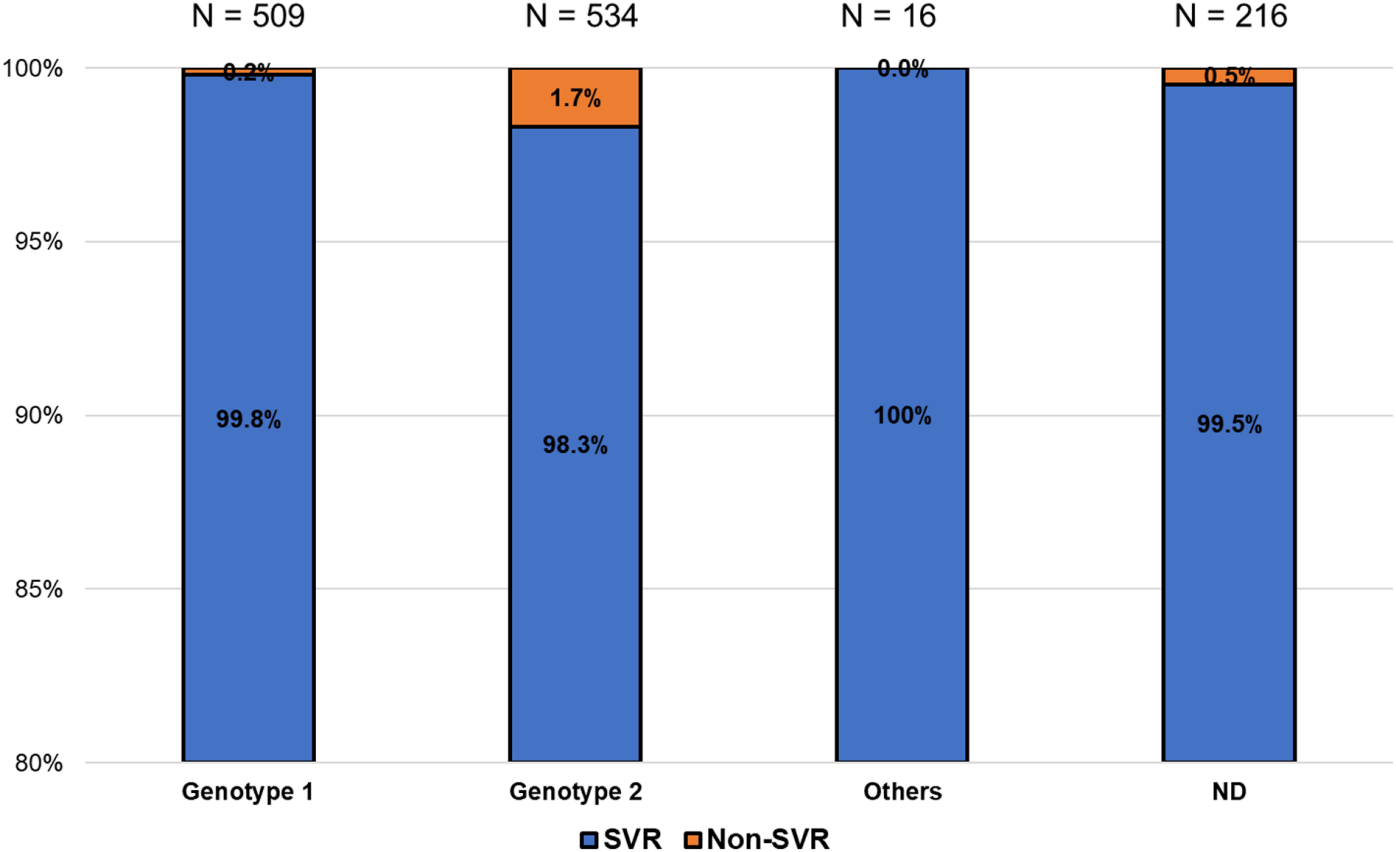
SVR rate for different genotypes.

The association between genotype and SVR rate was investigated. The SVR rates in patients with genotypes 1, 2, others, and not determined (ND) were 99.8% (508/509), 98.3% (525/534), 100% (16/16), and 99.5% (215/216), respectively, and no significant difference in the SVR rate was found among the genotypes (*P* = 0.6, Fig. 1).

### Impact of liver fibrosis status and treatment periods on the SVR

**Figure 2:**
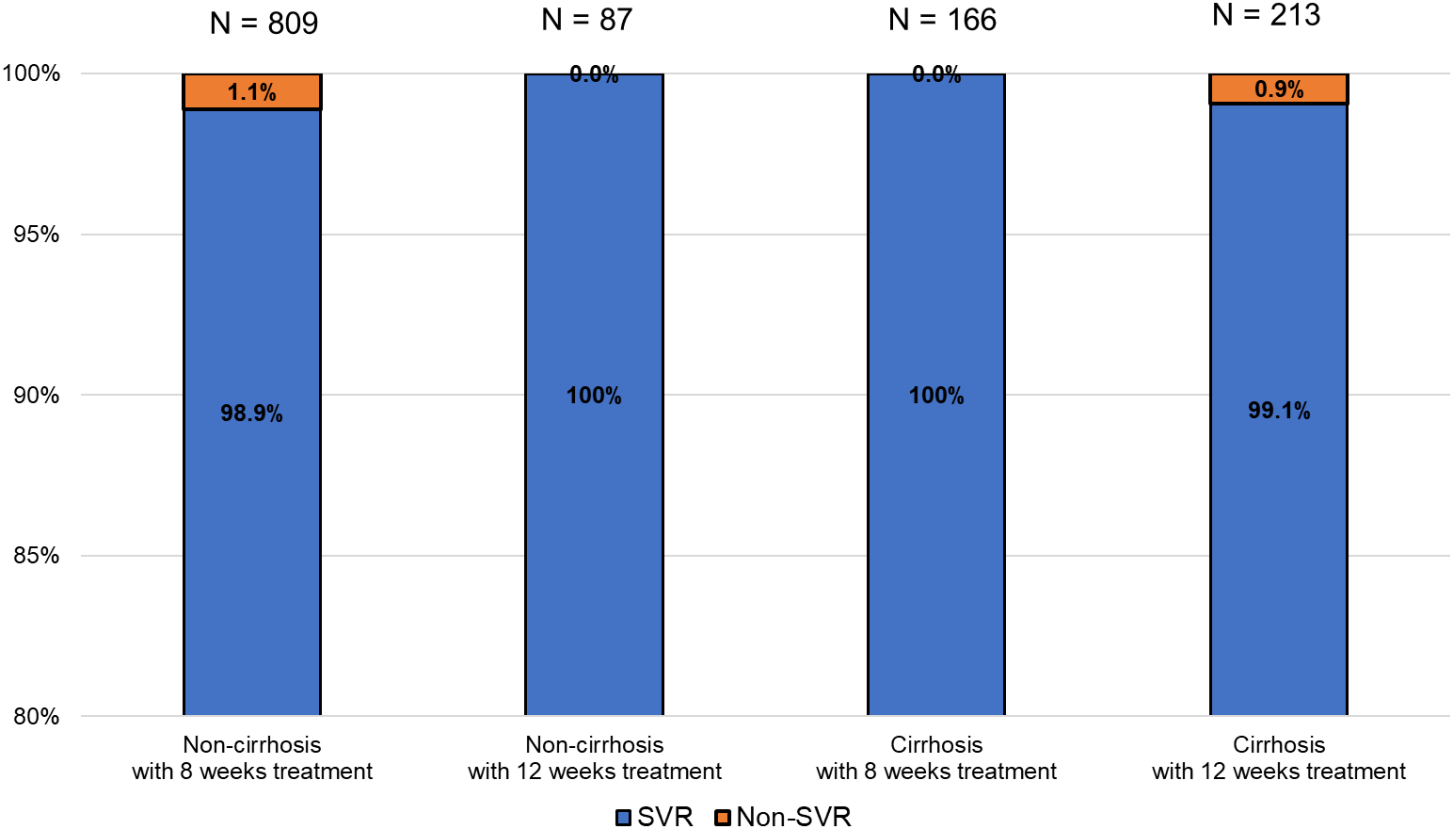
Impact of liver fibrosis status and treatment periods on the SVR.

Patients were stratified based on liver fibrosis status and treatment periods. Among the patients with chronic hepatitis (non‐cirrhosis), 809 received 8 weeks of treatment and 87 received 12 weeks of treatment. Among patients with cirrhosis, 213 received 12 weeks of treatment and 166 received 8 weeks of treatment. The characteristics of cirrhotic patients with 12 or 8 weeks of treatment are shown in Table 2. The median age was 74 and 76 years in patients with 12 and 8 weeks of treatment, respectively, with no significant difference between the two groups. AST and ALT levels were equal between the two groups, but albumin levels were significantly higher in patients with 8 weeks treatment, and platelet counts were significantly lower in patients with 8 weeks of treatment. The SVR rates in patients with chronic hepatitis with 8 weeks of treatment, chronic hepatitis with 12 weeks of treatment, cirrhosis with 8 weeks of treatment, and cirrhosis with 12 weeks of treatment were 98.9% (800/809), 100% (87/87), 100% (166/166), and 99.1% (211/213), respectively (Fig. 2). The SVR rate was not different among these groups (*P* = 0.4), and even patients with cirrhosis with 8 weeks of treatment could achieve an SVR rate of 100%.

**Table 2:** Baseline characteristics of patients with cirrhosis

Patients with cirrhosis (FIB‐4 ≥3.25)	12 weeks of treatment	8 weeks of treatment	
Number	213	166	*P*‐value
Age, years	74 (66–81)	76 (68–83)	0.2
Gender, %			
Female	117 (54.9)	104 (62.7)	0.1
Male	96 (45.1)	62 (37.3)	
Genotype, %			
1	88 (41.3)	68 (41.0)	0.2
2	84 (39.4)	64 (38.4)	
Others	5 (2.3)	0 (0)	
ND	36 (16.9)	34 (20.5)	
History of HCC, %	51 (23.9)	4 (2.4)	<0.001
Albumin, g/dL	3.8 (3.5–4.1)	4.0 (3.8–4.2)	<0.001
AST, IU/mL	56 (39–78)	54 (36–106)	0.5
ALT, IU/mL	47 (26–75)	43 (24–105)	0.9
Platelet counts, ×10^4^/μL	10.6 (8.3–13)	14.2 (12–17)	<0.001

*P*‐value indicates the comparison between 8 and 12 weeks of treatment.

Continuous data are shown in median (interquartile range).

ALT, alanine aminotransferase; AST, aspartate aminotransferase; HCC, hepatocellular carcinoma; ND, not determined.

### SVR rate in late responders

**Figure 3:**
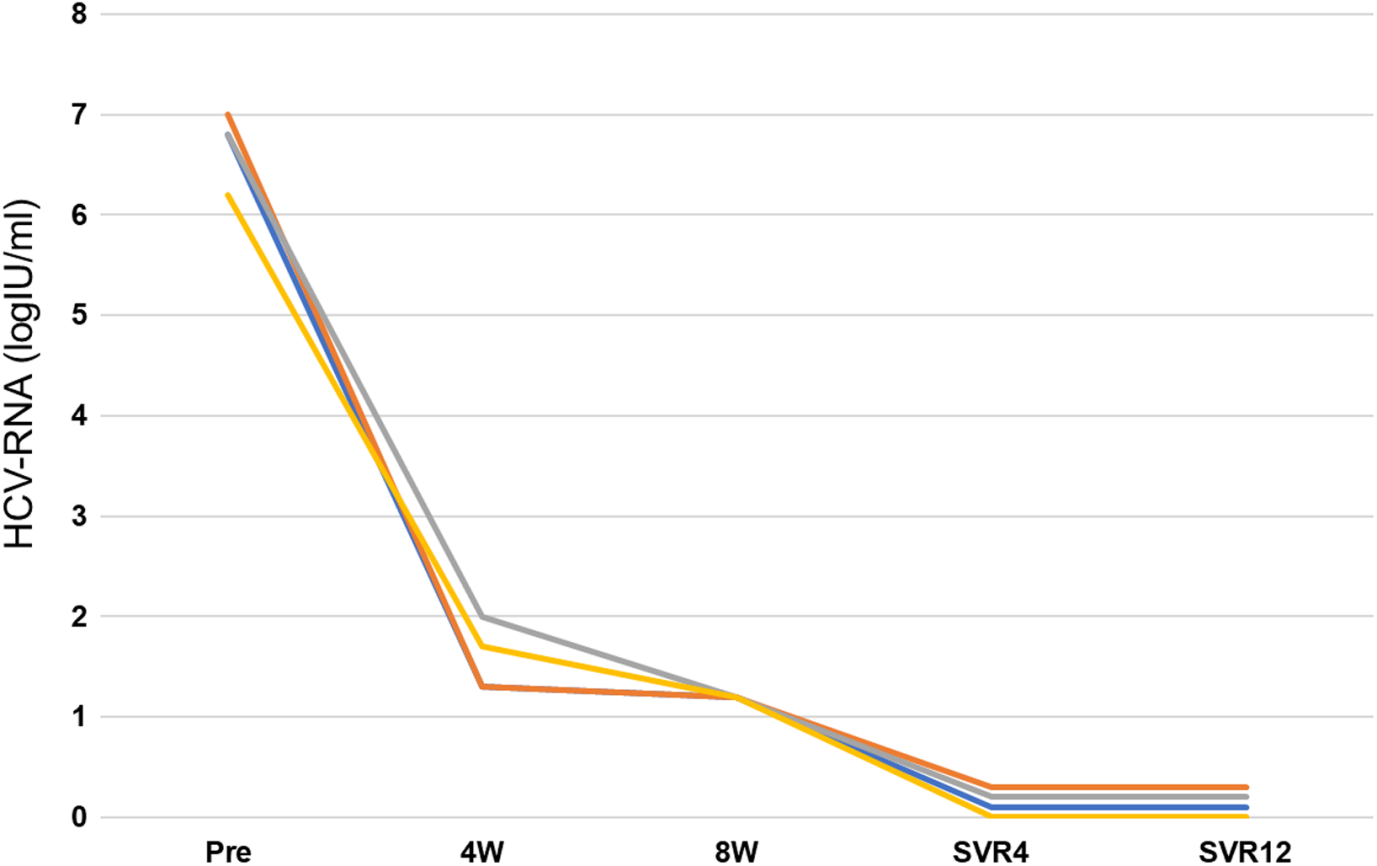
SVR in late responders.

Among the patients with 8 weeks of treatment, four had HCV‐RNA positivity (<1.2 log IU/mL) at 8 weeks (late responder). After stopping the treatment at 8 weeks, all these patients achieved SVR4 and SVR12 (Fig. 3).

### Adverse events

Symptomatic adverse events included pruritus in 139 (10.9%) patients, and fatigue, headache, and decreased appetite in 64 (5.0%), 35 (2.7%), and 21 (1.6%) patients, respectively. With regard to laboratory data, grade 2 and 3 bilirubin elevations were observed in 21 (1.6%) and 2 (0.2%) patients, respectively, but the bilirubin elevation improved in all patients during the natural course of the treatment.

## Discussion

### Main findings

This prospective, nationwide, multicenter cohort study demonstrated that GLE/PIB therapy for chronic hepatitis C had high efficacy (>98% SVR rate) regardless of liver fibrosis status and treatment periods. Even patients with cirrhosis receiving 8 weeks of treatment also achieved high SVR rates (100%). Therefore, the treatment periods for GLE/PIB therapy could be chosen with available modalities by each physician, and a high SVR rate could be achieved regardless of the decision.

### In context with published literature

Several DAA therapies have been developed and used in clinical practice, with all drugs showing high efficacy (>95% SVR rate). Currently, GLE/PIB is recommended as the first‐line treatment in JSH guidelines.^21^ As an advantage, GLE/PIB could be completed with only 8 weeks of treatment in patients with chronic hepatitis (non‐cirrhosis), whereas other drugs need 12 weeks of treatment. This advantage is beneficial for medication adherence and economic burden of HCV treatment. In Japan, 8 weeks of GLE/PIB therapy in patients with chronic hepatitis and 12 weeks in those with cirrhosis are permitted and reimbursable. However, one difficulty is the assessment of liver fibrosis. Although liver biopsy is the gold standard for the assessment of liver fibrosis, it has several limitations such as invasiveness or inter‐ and intra‐observer discrepancy.^24^ Several noninvasive modalities have been developed and used in clinical practice; however, accurately distinguishing between chronic hepatitis and cirrhosis is difficult.^25^, ^26^, ^27^, ^28^, ^29^, ^30^ Therefore, whether 8 weeks of treatment for cirrhosis may reduce treatment efficacy remains a concern. In this study, GLE/PIB therapy had high efficacy regardless of liver fibrosis status and treatment periods, and even patients with cirrhosis and 8 weeks of treatment achieved high SVR rates. In Western countries, 8‐week GLE/PIB therapy is implemented even in patients with cirrhosis, and a high SVR rate is observed.^31^, ^32^, ^33^ This result also supports our findings. Therefore, even if liver fibrosis status is underestimated in patients with cirrhosis (as defined by FIB‐4 >3.25) and shorter treatment periods (8 weeks) are chosen, high efficacy could be expected.

### Strengths and limitations

The major strengths of this study include its prospective, nationwide, multicenter cohort design and inclusion of over 1000 patients. However, treatment periods were chosen according to the decision of each physician. Furthermore, FIB‐4 was used as the criterion for cirrhosis in this study. Therefore, liver fibrosis status might not have been assessed adequately. However, these settings reflect real‐world clinical practice, and a high efficacy of GLE/PIB therapy could be observed in this setting. Since the SVR rate in patients with cirrhosis (as defined by FIB‐4 >3.25) and 8 weeks of treatment may be overestimated because of the selection bias for choosing treatment periods by physicians and the possibility of including non‐cirrhosis cases in the group, a further study with biopsy‐based fibrosis assessment is needed.

### Future implications

Data on the effect of liver fibrosis status and treatment periods on the efficacy of GLE/PIB therapy are limited. In this study, GLE/PIB therapy demonstrated high efficacy regardless of liver fibrosis status and treatment periods. Even patients with cirrhosis (as defined by FIB‐4 >3.25) and 8 weeks of treatment achieved high SVR rates. Our results indicate that liver fibrosis status could be evaluated using available modalities including blood test‐based markers, elastography, or liver biopsy, and physicians could choose periods of GLE/PIB therapy based on the results.

## Conclusion

In conclusion, GLE/PIB therapy for chronic hepatitis C had high efficacy regardless of liver fibrosis status and treatment periods. Periods of GLE/PIB therapy could be chosen with available modalities, and high SVR rates could be achieved regardless of the decision.

## Supporting information


**Data S1.** Supporting Figures.
