# Supplementary figures and images for: Effect of treatment periods on efficacy of glecaprevir and pibrentasvir in chronic hepatitis C: A nationwide, prospective, multicenter study
Source: JGH Open. 2024 Apr 25;8(4):e13068. doi: 10.1002/jgh3.13068 (PMC11046085; doi:10.1002/jgh3.13068)

## Slide 1
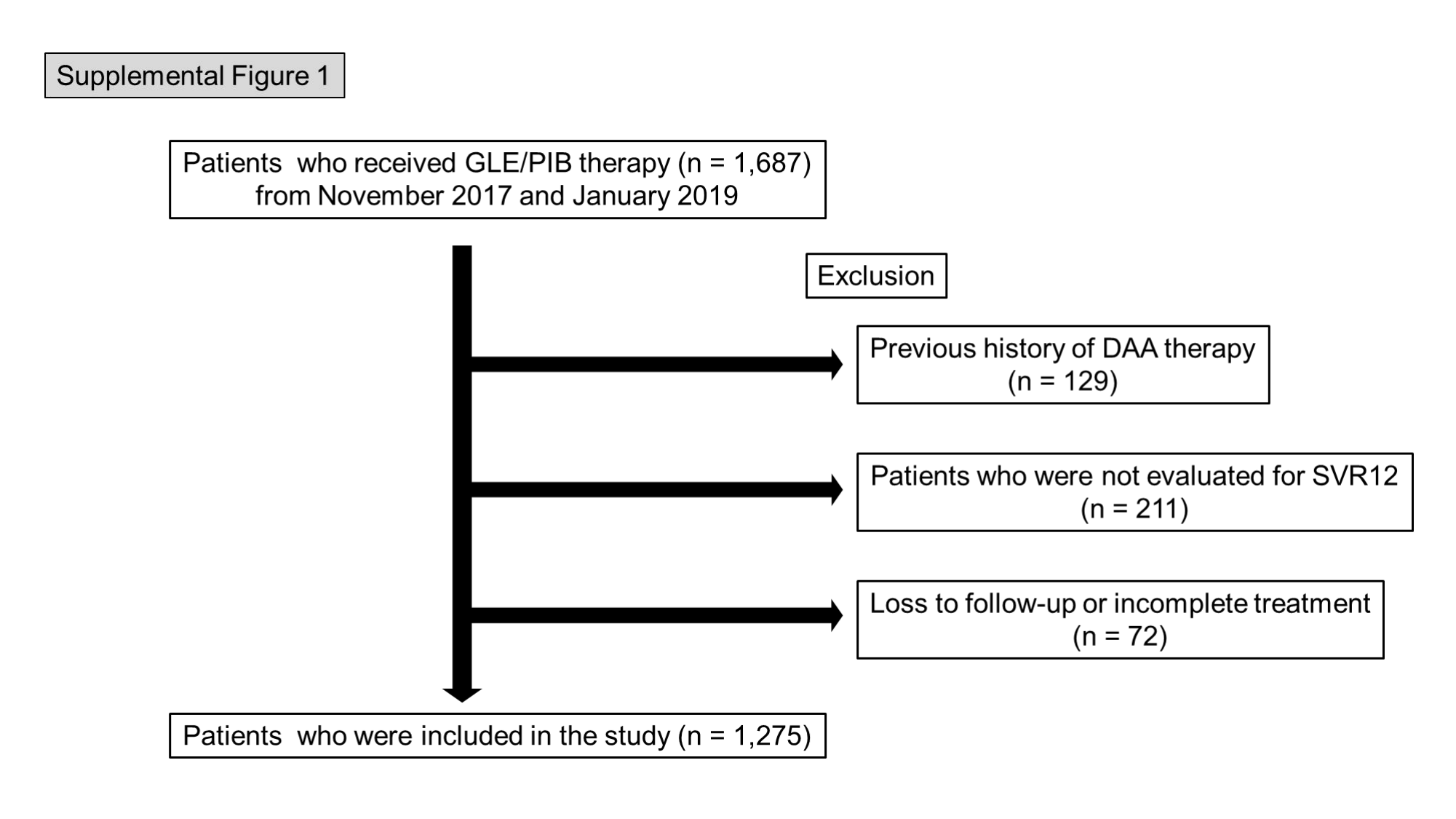

Supplement: Supplementary file 1 — Data S1. Supporting Figures. [file JGH3-8-e13068-s001.pptx]
